# Asymptomatic Candiduria due to *Candida inconspicua* in a Patient With Hematologic Malignancy

**DOI:** 10.1155/crdi/1373865

**Published:** 2026-01-29

**Authors:** Andrés Soto, Javier Tinoco-Cahuana, Paulo Charpentier-Videla, Edgardo Rojas-Mancilla, Diego Macias-Cofre, Daniela Caceres-Canales, Cecilia Morales-Gonzalez, Lissette Guajardo-Quileñan, Jose Luis Briones-Martinez, Esteban Mejias-Escobar, Carolina Selman-Bravo, Francisca Sanchez-Jorquera

**Affiliations:** ^1^ Fundación Arturo López Pérez OECI Cancer Center, Unit of Infectology, Santiago, Chile, falp.cl; ^2^ Facultad de Medicina, Universidad de Chile, Santiago, Chile, uchile.cl; ^3^ Fundación Arturo López Pérez OECI Cancer Center, Clinical Laboratory, Santiago, Chile, falp.cl; ^4^ Escuela de Terapia Ocupacional, Facultad de Salud y Ciencias Sociales, Universidad de Las Américas, Sede Providencia, Santiago, Chile, udla.edu.ec

**Keywords:** *Candida inconspicua*, Candiduria, hemato-oncological, opportunistic pathogen

## Abstract

*Candida inconspicua* is an uncommon opportunistic yeast, increasingly reported in immunocompromised patients. We describe a case of asymptomatic Candiduria caused by *C. inconspicua* in a 43‐year‐old woman with Philadelphia chromosome‐positive B‐cell acute lymphoblastic leukemia undergoing chemotherapy. During multiple episodes of high‐risk febrile neutropenia, urine cultures repeatedly isolated *C. inconspicua*, identified via MALDI‐TOF MS and exhibiting high fluconazole resistance (MIC: 16.0 μg/mL). Despite these findings, no antifungal therapy was administered due to the absence of urinary symptoms and resolution of fever with antibacterial treatment. This case illustrates the clinical dilemma posed by rare, drug‐resistant *Candida* species in asymptomatic infections, emphasizing the importance of precise microbiological identification and antimicrobial stewardship. The emergence of *C. inconspicua*, with potential for resistance and biofilm formation, underscores the need for careful evaluation in hemato‐oncological patients, particularly when considering the risks and benefits of initiating antifungal therapy in the absence of clinical symptoms.

## 1. Introduction

*Candida inconspicua*, formerly known as *Torulopsis inconspicua*, is part of the microbiota of the oropharynx, respiratory tract, and vagina [1, 2]. Candiduria is more frequently observed in hospitalized patients compared with outpatients and is often associated with respiratory tract colonization in immunocompromised individuals, such as cancer patients [1, 3, 4]. The global incidence of *C. inconspicua* is rare but recurrent [5]. The presence of yeast in urine is associated with increased morbidity and mortality in patients with underlying conditions, highlighting the need for accurate diagnosis [6]. Treatment of Candiduria remains controversial. Current guidelines recommend antifungal therapy primarily for symptomatic cases or those at high risk of developing candidemia [7–9]. Therefore, precise species identification and determination of antifungal sensitivity are pivotal for clinical decision‐making [10, 11].

Here, we present a case of asymptomatic Candiduria caused by *C. inconspicua* in a hemato‐oncological patient. This case highlights the challenges of accurately managing asymptomatic fungal infections and the fluconazole resistance exhibited by *C. inconspicua*.

## 2. Case Presentation

A 43‐year‐old woman diagnosed with Philadelphia chromosome–positive acute B‐cell lymphoblastic leukemia began a second cycle of ambulatory chemotherapy under the R‐hyper‐CVAD regimen, combined with dasatinib, a tyrosine kinase inhibitor, for aplasia management in September 09, 2023 (Day 0; Table 1). Renal and hepatic functions were assessed prior to chemotherapy and found to be within normal ranges. Furthermore, the creatinine level was 1.2 mg/dL and liver function tests were normal. Two weeks later, the patient was admitted due to a severe neutropenia, confirmed by an absolute neutrophil count of 0 cells/μL and a platelet count of 24,000 cells/μL (Day 12). The patient rapidly developed a fever up to 37.9°C, chills, and an isolated episode of hematuria with clot passage, without other symptoms. Physical examination revealed abdominal pain in the right hemiabdomen, without signs of peritoneal irritation. Previously, in August 2023, she had been admitted for high‐risk febrile neutropenia (HRFN) during a chemotherapy, which resolved with ertapenem and amikacin treatment.

**TABLE 1:** Clinical timeline of clinical events, diagnostic findings, interventions, and follow up.

Date	Event	Absolute neutrophil count
09.09 (Day 0)	Started R‐hyper‐CVAD chemotherapy with dasatinib.	1500
21.09 (Day 12)	Severe neutropenia with fever, hematuria, abdominal pain.	0
21.09 (Day 12)	Began ertapenem, amikacin, transfusion, anidulafungin prophylaxis.	0
21.09 (Day 12)	CT showed enterocolitis and kidney inflammation.	0
22.09 (Day 13)	Urine culture > 100,000 CFU yeasts, elongated forms.	220
23.09 (Day 14)	MALDI‐TOF identified *Candida inconspicua*.	660
23.09 (Day 14)	Susceptibility: fluconazole resistant, others susceptible.	660
28.09 (Day 19)	Blood cultures remained negative.	1580
29.09 (Day 20)	Fever resolved, ANC recovered, discharged.	4650
13.11 (follow‐up)	Two HRFN episodes, urine positive for *C. inconspicua*.	0
07.12 (follow‐up)	No antifungals, favorable outcome.	0
09.01 (follow‐up)	Urine culture negative	0

In order to evaluate possible bacteremia or bacteriuria, laboratory tests were performed through aerobic peripheral blood cultures and urine culture, respectively, and treatment with ertapenem and amikacin was initiated, along with transfusion support. A midstream urine sample was collected in a sterile container, and culture was performed using a calibrated 1 μL loop on Chromo UTI and CNA media. The direct microscopic examination revealed structures consistent with yeasts, leading to subsequent subculturing onto Chromo UTI, blood agar, and chocolate agar for isolation and identification.

Additionally, the patient received primary antifungal prophylaxis with anidulafungin, starting with a loading dose of 200 mg followed by 100 mg daily until the absolute neutrophil count exceeded 500 cells/μL. A computed tomography scan of the thorax, abdomen, and pelvis revealed an inflammatory process in the ascending colon and left kidney with nephrolithiasis. A gastrointestinal focus of HRFN was considered, with probable neutropenic enterocolitis.

**FIGURE 1:**
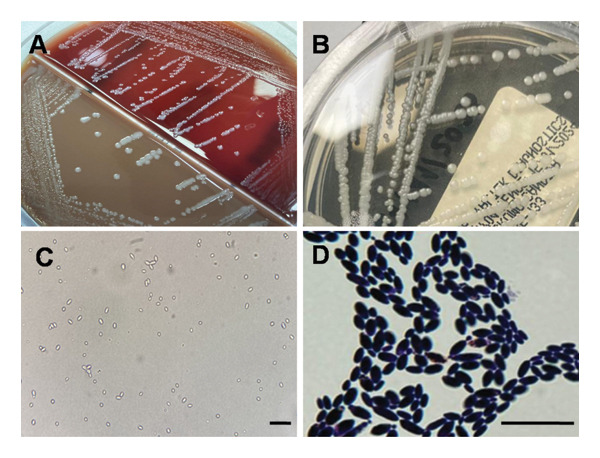
Isolation of *Candida inconspicua* in urine culture. (A) White, creamy, smooth, round, and raised colonies on Columbia blood agar/chocolate agar plate (Valtek S.A., Santiago, Chile). (B) Creamy, smooth, round, and raised colonies without pigmentation on chromogenic *Candida* agar (Valtek S.A., Santiago, Chile). (C) Direct observation of a negative germ tube test. (D) Gram stain of elongated yeasts observed at 1000x magnification. Scale bar: 10 μm.

The urine culture on Columbia blood agar/chocolate agar plate showed white, creamy, smooth, round, and raised colonies, with a count exceeding 100,000 CFU/mL (Figure 1(A)). The urinary sediment was noninflammatory but revealed the presence of elongated yeasts observed at 1000x magnification (Day 13, Figure 1(D)). Additionally, chromogenic candida agar displayed creamy, smooth, round, and raised colonies without pigmentation (Day 14, Figure 1(B)). Also, the germ tube test was negative under direct observation (Figure 1(C)). Blood cultures incubated in the automated BACT/ALERT system (bioMérieux, France) remained negative at Day 19. Identification was positive for *C. inconspicua*, confirmed by matrix‐assisted laser desorption ionization time‐of‐flight mass spectrometry (MALDI‐TOF MS; MALDI‐TOF VITEK MS PRIME; bioMérieux, France) on Day 14. An in vitro susceptibility study, performed by broth microdilution using the Sensititre Yeast One system (Thermo Fisher Scientific, USA), showed minimum inhibitory concentrations (MICs) of 16.0 μg/mL for fluconazole, 0.12 μg/mL for voriconazole, 0.03 μg/mL for caspofungin, ≤ 0.015 μg/mL for anidulafungin, and 0.25 μg/mL for amphotericin B (Day 14). There are currently no established Clinical and Laboratory Standards Institute (CLSI) or EUCAST guidelines for MIC interpretation of *C. inconspicua*. Thus, it was conducted comparatively utilizing reference ranges from related species including *Candida glabrata* and *Candida krusei*, as suggested in the literature [2, 5]. These values presented serve as indicative microbiological and epidemiological data rather than conclusive clinical classification.

Following antimicrobial treatment, the patient showed resolution of both fever and abdominal pain, without new episodes of hematuria and with recovery of the absolute neutrophil count. Upon evaluation by the Infectious Diseases Unit, it was determined that the *C. inconspicua* isolated from the urine culture did not have a pathogenic role and, therefore, specific antifungal treatment was not initiated. On Day 20, the patient was discharged from the hospital after completing 7 days of ertapenem therapy; amikacin had been discontinued 48 h after admission. During November and December 2023, the patient experienced two additional episodes of HRFN with a gastrointestinal focus, again presenting positive urine cultures for *C. inconspicua* without urinary symptoms. Repeated urine cultures consistently isolated *C. inconspicua* with the same susceptibility profile. No antifungal therapy was initiated during these episodes, and the patient showed a favorable clinical evolution without complications attributable to fungal infection.

The patient had multiple follow‐up urine cultures following the initial diagnosis, all of which consistently yielded *C. inconspicua* (Table 1). Additionally, imaging studies were conducted for recurrent febrile neutropenia, all of which did not reveal inflammatory abnormalities in the urinary tract indicative of *Candida* urinary infection. This follow‐up indicates that, despite ongoing colonization, there was no progression to symptomatic or invasive disease.

## 3. Discussion

*C. inconspicua*, first described as *Torulopsis inconspicua* in 1952 [12] and reclassified as *Candida* in 1978 [13, 14], is an uncommon but clinically significant yeast, particularly in immunocompromised individuals. While it is often isolated from the gastrointestinal and respiratory tracts of patients with cancer, diabetes mellitus, or HIV, its presence in Candiduria is rare [3, 4]. Despite this rarity, *Candida* spp. remains the predominant cause of invasive fungal infections both in Chile and worldwide [1, 5, 15, 16]. In Chile, a low prevalence of *C. inconspicua* have been reported, despite increasing global detection. A large Chilean study reported a single case of candidemia by *C. inconspicua* [17]. Furthermore, a review on clinical significance of uncommon *Candida* in South America did not report *C. inconspicua* [18].

Over the past decade, there has been a notable expansion in the spectrum of isolated *Candida* species, primarily due to the incorporation of new technologies such as MALDI‐TOF MS and genetic testing, which allow the identification of species rarely considered pathogenic in clinical isolates, with high performance and utility. These new species include *C. inconspicua* [19].

The reasons for the emergence of new species are not yet clear, but selective pressure from prophylactic management regimens based on azole derivatives has been suggested [9]. These rare species isolates can represent up to 10% of all *Candida* isolates as etiological agents in some centers. A multicenter study reported an increase in isolation rates from 2001 to 2004, rising from 9 to 276 reported cases [16, 20]. Furthermore, *C. inconspicua* has been described as a relevant agent in hemato‐oncological patients although its incidence is not well characterized. In these limited cases, previous colonization by *C. inconspicua* has been associated with the subsequent development of clinical infections, such as candidemia [4].

Macroscopically, *C. inconspicua* appears as creamy, white, medium‐sized colonies on chocolate blood agar. On selective and chromogenic agar, whitish colonies are observed. In the Gram staining, they appear as elongated yeasts [13]. Like other *Candida* species, *C. inconspicua* has the ability to form biofilms in mucosa and medical devices, including catheters, favoring long‐term persistence [21].

In this case report, *C. inconspicua* showed a high MIC for fluconazole, indicating resistance. *C. inconspicua* is among the emerging species resistant to fluconazole, frequently isolated as a cause of infection [4]. Depending on the origin of the strain, fluconazole resistance can range from 26% (skin and soft tissues) to 62% (genital tract), suggesting high phenotypic heterogeneity among *C. inconspicua* isolates. The genomic plasticity exhibited by *C. inconspicua* may correlate with greater phenotypic diversity and a higher propensity to adapt to antifungal drugs and develop new resistances [20]. Another consideration is that CLSI does not include MIC breakpoints for this species in its standards for in vitro susceptibility interpretation [7]. Deciding whether to treat Candiduria can be controversial, and currently, the prevailing criterion is to administer antifungal treatment in cases where the patient shows symptoms and is at a high risk of developing candidemia [7–9]. Therefore, precise species identification and determination of antifungal sensitivity are the key for effective treatment [10, 11].

The effective clinical management and understanding of the epidemiology of fungal infections rely on accurate identification of yeast strains. In this regard, *C. inconspicua* must be considered an opportunistic pathogen in immunocompromised patients, and its identification may present particular challenges in terms of treatment [3, 4, 9]. Current recommendations focus on reducing unnecessary antifungal administration. Moreover, with the increasing reports of fluconazole resistance in this *Candida* species, echinocandins are currently the first choice for treatment [5]. Recent antifungal stewardship interventions have demonstrated improvements in antifungal consumption, suggesting that restricting antifungal therapy can improve patient outcomes [22].

*C. inconspicua* can persist asymptomatically in the urinary tract even among immunosuppressed patients. This species is becoming increasingly isolated globally and exhibits low susceptibility to fluconazole. However, its pathogenicity depends on host factors and transition from colonization to invasive disease. Colonization represents a critical virulence factor, with isolates frequently derived from the digestive and respiratory tracts rather than genuine urinary tract infection [4]. Thus, urinary isolation in neutropenic hosts may still represent colonization rather than clinically active disease [1, 4, 5].

Despite the patient presented fluconazole‐resistant *C. inconspicua*, antifungal therapy was not administered regarding the lack of urinary symptoms and clinical improvement. Current international guidelines endorse this approach, notably the 2025 ECMM/ISHAM/ASM global guideline on candidiasis, which explicitly advices against treating asymptomatic Candiduria in hematology or transplant patients, highlighting the necessity of minimizing unnecessary antifungal exposure [23]. Available evidence shows that the progression of Candiduria to candidemia is low, estimated between 1% and 8%, even in high‐risk populations [24]. This low conversion rate reinforces the principle of minimizing treatment. Consequently, our management adhered to evidence‐based recommendations, emphasizing clinical stability rather than microbiological results. This case points out the importance of personalized decisions in antifungal stewardship programs, especially for immunocompromised patients.

In our case, the recurrent isolation of *C. inconspicua* without urinary symptoms or systemic decline justified a conservative approach that avoided unnecessary antifungal exposure while ensuring close monitoring. This outcome highlights the importance of precise, risk‐based antifungal stewardship, underscoring that careful clinical assessment can safely guide management even when rare, resistant *Candida* species are detected.

## 4. Conclusions

Persistent urinary tract colonization by an antifungal‐resistant yeast such as *C. inconspicua* represents a sustained clinical challenge, as immunosuppression related to future intensive chemotherapy cycles may facilitate progression from colonization to invasive infection. In this context, therapeutic options are limited by intrinsic fluconazole resistance and the low renal excretion and reduced effectiveness of echinocandins in *Candida* urinary tract infections. Moreover, the potential need for invasive urological procedures in patients with asymptomatic Candiduria increases the risk of mucosal translocation and systemic dissemination, particularly in the presence of concomitant neutropenia. These considerations underscore the importance of accurate strain identification, structured follow‐up, close clinical surveillance, and preventive strategies, along with individualized therapeutic planning, including the use of antifungal agents such as amphotericin B or voriconazole when clinically indicated. Overall, *C. inconspicua* should be regarded as a clinically relevant opportunistic pathogen in this setting.

## Author Contributions

A.S.: writing–original draft, writing–review and editing, investigation, formal analysis, conceptualization, and data curation. J.T‐C.: writing–review and editing, investigation, formal analysis, and data curation. P.C‐V.: writing–review and editing, investigation, formal analysis, and data curation. E.R‐M.: writing–review and editing, investigation, methodology, and formal analysis. D.M‐C.: investigation, formal analysis, and data curation. D.C‐C.: investigation, formal analysis, and data curation. C.M‐G.: writing–original draft, writing–review and editing, and formal analysis. L.G‐Q.: writing–original draft, writing–review and editing, and formal analysis. J.L.B‐M.: writing–original draft, writing–review and editing, and formal analysis. E.M‐E.: writing–original draft and writing–review and editing. C.S‐B.: writing–review and editing, supervision, and conceptualization. F.S‐J.: writing–review and editing, supervision, and conceptualization.

## Funding

There was no specific funding for this case report.

## Ethics Statement

Written informed consent was obtained from the patient for publication of this case report, in accordance with local ethics committee requirements. The local ethics committee reviewed the case antecedents and approved the dissemination of the information.

## Conflicts of Interest

The authors declare no conflicts of interest.

## Data Availability

The data that support the findings of this study are available on request from the corresponding author. The data are not publicly available due to privacy or ethical restrictions.
